# Estimating densities of larval Salmonflies (*Pteronarcys californica*) through multiple pass removal of post-emergent exuvia in Colorado rivers

**DOI:** 10.1371/journal.pone.0227088

**Published:** 2020-04-10

**Authors:** Brian D. Heinold, Dan A. Kowalski, R. Barry Nehring

**Affiliations:** 1 Colorado Parks and Wildlife, Fort Collins, Colorado, United States of America; 2 Colorado Parks and Wildlife, Montrose, Colorado, United States of America; Duke University Marine Laboratory, UNITED STATES

## Abstract

Traditional methods of collecting, sorting, and identifying benthic macroinvertebrate samples are useful for stream biomonitoring and ecological studies, however, these methods are time consuming, expensive, and require taxonomic expertise. Estimating larval densities through collection of post-emergent exuvia can be a practical and time efficient alternative. We evaluated the use of multiple pass depletion techniques of the post-emergent exuvia of *Pteronarcys californica* to estimate larval densities at ten sites in three Colorado rivers. Exuvia density was highly correlated with both final-instar larval density (R^2^ = 0.90) and total larval density (R^2^ = 0.88) and the multiple pass removal technique performed well. Exuvia surveys found *P*. *californica* at three low density sites where benthic sampling failed to detect it. At moderate and high density sites the exuvia surveys always produced lower density estimates than benthic surveys. Multiple pass depletion estimates of exuvia proved to be an accurate and efficient technique at estimating larval densities and provided an effective alternative for traditional benthic sampling when objectives are detecting and monitoring *P*. *californica*, especially at low density sites.

## Introduction

Evaluating the condition of freshwater ecosystems through benthic macroinvertebrate communities is a common approach for stream health assessment and biomonitoring [1–3]. These methods characterize and compare aquatic invertebrate communities among sites using regionally developed standards. Benthic studies, while useful, are labor and time intensive, expensive, sensitive to sampling techniques, and require taxonomic expertise. The costs can be justified by the valuable data used by government agencies, researchers, and water managers to monitor water quality and to describe and understand the function of river ecosystems. But, if sampling objectives are more specific and budgets are limited, whole community benthic sampling may not be necessary or the most appropriate technique.

One ecologically important aquatic invertebrate commonly used as a bioindicator is the Giant Salmonfly (*Pteronarcys californica* Newport). It is useful for biomonitoring because of its sensitivity to habitat alteration, widespread distribution in western North America [4, 5], multi-year larval life stage, large body size, easy identification, low larval dispersal, and well defined larval habitat preferences [6–9]. *Pteronarcys californica* is among the largest and longest lived stonefly in western North America [10–12]. In Colorado, larvae typically inhabit unpolluted, medium to large, permanent streams with unconsolidated cobble and large gravel substrates between 1,500 and 2,500 m in elevation [13, 14]. Adults emerge from late May to early July and recruitment begins in April after a 9–10 month egg diapause [15] followed by a three to four year aquatic larval stage [16, 17]. Mature larvae (larvae expected to hatch that year) migrate toward the stream bank to stage a highly synchronous adult emergence. Salmonflies typically emerge at night crawling out of the water onto riparian substrates to become winged terrestrial adults where they leave post-emergent exuvia (hereafter, exuvia).

*Pteronarcys californica* plays an important ecological role, both in biomass and abundance, in stream and riparian food webs. As shredders, larvae process coarse organic matter like vascular plants and algae [9, 18] making the nutrients available to other feeding groups as detritus or body biomass [19]. Salmonflies can comprise a large portion of the benthic biomass because of their large body size and high densities in suitable habitat [20, 21], making them an important component of stream food webs for crayfish, other invertebrates, and trout [22, 21]. Terrestrial adults are part of a critical link for aquatic-riparian nutrient and energy exchange [23] as prey for frogs, birds, bats, and spiders [15, 24]. Despite its ecological importance, range-wide declines of *P*. *californica* have been documented in the Logan and Provo Rivers in Utah [25, 26], at least four rivers in Montana [27], and in the Gunnison and Colorado Rivers in Colorado [4, 28] likely due to the effects of large main stem impoundments like changes in flow and temperature regimes, decreased water quality, and flow depletions.

Density of benthic macroinvertebrates is traditionally estimated by systematically collecting samples from a fixed area of the stream bed. Alternative methods have been recently developed to indirectly survey communities by identifying and enumerating exuvia. These methods can reduce time and labor of traditional techniques while providing reliable population density estimates, community structure, and life history information. Ruse [29] inferred the community structure of chironomids from larval and pupal exuvia and Foster and Soluk [30] estimated densities of the endangered Hine’s emerald dragonfly (*Somatochlora hineana*) more accurately by sampling larval exuvia than by collecting adults. Raebel et al. [31] stated the importance of exuvia collections to avoid bias in adult Odonata surveys. DuBois [32] enhanced these studies by using a depletion population estimator to approximate exuvia densities and detection probabilities of Anisoptera. Richards et al. [33] correlated *P*. *californica* exuvia densities and live (wet) larval body weights with substrate embeddedness to demonstrate differences in life history, distribution, and abundance above and below a main stem impoundment. Their work provided a foundation in the development of our novel technique to estimate larval densities through multiple pass removal sampling of exuvia.

Multiple pass removal sampling is a commonly used technique in wildlife and fisheries to estimate population size of closed populations. Assumptions of the model used to analyze these data are closure (no deaths, births, emigration, or immigration) and constant capture probability [34, 35] that must be met to avoid bias [36, 37]. If more than two depletion events are completed then assumptions about capture probabilities can be relaxed and capture rates for different passes can be estimated [35]. When populations can be considered geographically and demographically closed (due to isolation or short sampling time period) then population estimation can be accomplished rather simply if good unbiased estimates of detection probability are possible [35].

The objective of this study was to compare traditional benthic invertebrate sampling with multiple pass removal techniques to evaluate if closed population estimation models can be used to estimate the density *P*. *californica* larvae. We evaluated this by correlating density estimates of shed exuvia from multiple pass removal models from the riparian area with densities of larvae from benthic samples.

## Materials and methods

### Ethics statement

No vertebrate organisms were involved in this study that were covered by an institutional animal care and use committee. No state or federally endangered species was involved in the study. The ten study sites included six sites on public land; site 1 on Town of Granby Kaibab Park, site 4 on Colorado Parks and Wildlife Hot Sulphur Springs State Wildlife Area, and sites 5, 8, 9, and 10 were on Bureau of Land Management property. No specific permissions were required for these locations and research activities but all of the managing entities were coordinated with during the study. Sites 2, 3, 6, and 7 were on private land and permission was obtained for all locations and activities of the study.

### Study area

**Fig 1:**
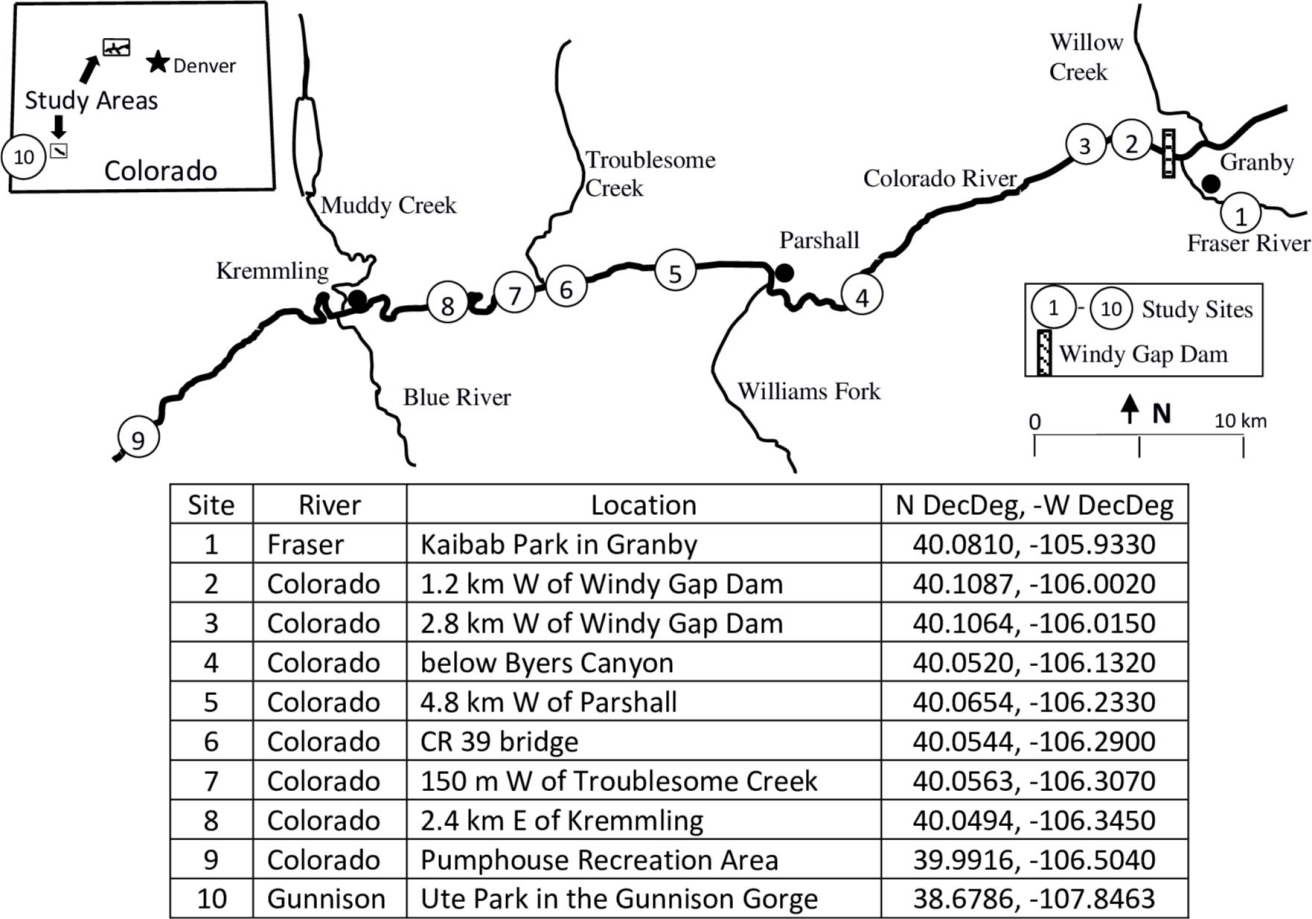
Benthic and exuvia collection sites of *Pteronarcys californica* in 2010 from the Colorado and Fraser Rivers. Gunnison River site shown only on inset map.

We collected salmonfly larvae and exuvia density estimates at 10 sites on three rivers in western Colorado, USA. Eight study sites were on the Colorado River, one on a main tributary; the Fraser River, and one site was on the Gunnison River (Fig 1). These rivers are 5^th^ and 6^th^ order streams with pool‐riffle morphology in the headwaters of the Colorado River basin in the Rocky Mountains. The distance between site one on the Fraser River and site nine on the Colorado River is approximately 71 stream kilometers and sites range in elevation from 2,426 m to 2,119 m. Average wetted widths of the Colorado Rivers ranged from 21.6 m to 42.3 m. Average wetted width on the Fraser River site was 15.3 m. The Gunnison River site is at an elevation of 1,608 m and an average wetted width of 33.5 m. Average annual discharge of the Fraser and Colorado Rivers increases downstream from 4.2 m^3^/s at site one to 29.2 m^3^/s at site nine and the Gunnison River site averages 46.7 m^3^/s. All sites were located at riffle areas with gravel, cobble, and boulder substrates.

### Benthic sampling

Three benthic subsamples were taken at each site between 15–18 April 2010 from the Colorado and Fraser Rivers and 10 May 2010 from the Gunnison River, approximately one month prior to the typical adult emergence of *P*. *californica*. All sites were located in riffle areas dominated by cobble substrates interspersed with boulders and gravel. A modified Surber sampler with a 0.25 m^2^ sampling frame (55.0 cm x 45.5 cm) and 150 μm mesh net was used. Cobbles larger than 10 cm in diameter were individually scrubbed with a brush, invertebrates washed into the net, and then the cobbles removed from the sampling frame. Remaining substrate within the frame was disturbed to a depth of 10 cm to dislodge invertebrates into the net. Contents were preserved with 80% ethanol in 2 L plastic jars.

In the lab, all *P*. *californica* larvae were sorted, sexed [21, 38], and measured for total length (TL) from the anterior tip of head to the posterior tip of the epiproct to the nearest millimeter under a dissecting microscope with a calibrated ocular micrometer. Length frequency histograms for male and female larvae were separately constructed based on TL to separate annual year classes. Body size measurements can be used to separate cohorts of this merovoltine species due to the long lifespan, fast growth, and large size [16, 38, 39]. We based our approach on separating annual cohorts on Townsend and Pritchard [16] except we constructed length frequency histograms from total length measurements rather than head-capsule width and wing-pad length. The largest size cohort of both males and females was considered to be mature larvae, the final instar larvae expected to emerge in that year. Densities of mature larvae and densities of all larvae were calculated and used in separate analyses for correlation with exuvia densities.

### Exuvia sampling

Sampling began with the onset of *P*. *californica* adult emergence on the Colorado River at site nine on 2 June 2010 and proceeded upstream to end at site one on the Fraser River on 21 June 2010; sampling at site 10 on the Gunnison River lasted from 16–23 June 2010. Each site was sampled beginning on the day when the first exuvia was found or winged adults were observed and continued daily until exuvia were no longer found. Data collection was performed by searching for exuvia within a maximum width of 10 m of the bank along two 30.5 m transects on one side of the river. Exuvia sampling sites were on the stream banks directly adjacent to benthic sampling sites. Searching at each individual site extended from the water’s edge laterally until no more exuvia were found, the search distance varying by site, generally depending on the complexity of the shoreline and thickness of riparian vegetation. The average width searched was measured at each site. Collections at a site were conducted by two to four people completing two or three removal passes with identical effort and personnel on each pass. Specimens were taken only when attached to dry riparian and emergent substrates; none were taken from the water to avoid counting ones that possibly drifted into the site. Exuvia were enumerated using hand held counters, stored in sealable bags, and removed from the search area.

### Data analysis

Area of benthic habitat was estimated by multiplying the sampling section length (always 30.5 m) by the average wetted channel width derived from 10 evenly spaced cross-channel transects. To evaluate the appropriateness of this sampling technique and the assumptions of the removal model, three pass removal data were compared to two pass data for nineteen sampling events when sufficient numbers of exuvia were found. Multiple pass removal data analyzed with the Huggins Closed Capture model in Program MARK [40, 41]. The Huggins Closed Capture Model can be used to analyze removal data by fixing the recapture probability to zero [35]. This model gives identical estimates to simpler two pass removal models like the Zippin model [34] but can analyze more data types with more sophisticated methods. The simple two pass models can easily be calculated on a spreadsheet while the Huggins model in Program MARK allows for more complex analysis such as modeling capture probabilities that vary by pass or being influenced by individual covariates [35]. While the two pass removal sampling takes less time in the field to complete and is simple to analyze, estimates could be biased if the assumption of constant capture probability between the two passes is violated [37].

To evaluate if the simpler constant capture probability model was sufficient with these data, Program MARK was used to compare two models for all occasions where three pass removals were completed. One model used a single constant capture probability (identical to Zippin estimate) and the second varied capture probability by pass, allowing a different capture probability for the first pass than the second and the third passes. Declining capture probability with subsequent passes is a common source of bias of removal models in fisheries data [36, 37] and comparing the two models, the population estimates, and capture probabilities allowed us to evaluate the assumption of constant capture probability of the simpler two pass model. The two models were compared using the information theoretic approach [42]. The small sample size version of Akaike’s Information Criterion (AIC*c*) was calculated for each model and model weights were compared to judge the top model. To evaluate if exuvia densities accurately estimated larval densities, we used simple linear regression in Program R [43]. Exuvia densities were the dependent variable and densities of mature larvae and all age class larvae were used in separate analyses as independent variables. Because sites were visited multiple times to encompass the entire emergence, population estimates for a single site from multiple days were added together to get a final density estimate.

## Results

Adult emergence of *P*. *californica* lasted between 2–8 days at each site and proceeded upstream approximately 4 km per day. Early in the emergence, male exuvia were dominant and sex ratios were more even toward the end of the emergence. Approximately 97% of exuvia (n = 21,526) were collected within two meters of the bank. A total of 47 separate surveys were completed at the 10 sites in June 2010 and 19 of those surveys found enough exuvia for the full three pass removal sampling. A total of 592 larvae were collected during the benthic sampling. Larvae from the Colorado and Fraser Rivers separated into four year classes; mature female larvae were ≥39 mm TL (mean 46.5, SE 0.51) and males ≥ 35 mm TL (mean 39.2, SE 0.34) (Table 1). Larvae from the Gunnison River separated into three year classes; mature female larvae ≥41 mm TL (mean 49.1, SE 0.51) and mature males ≥ 37 mm TL (mean 41.9, SE 0.46) (Table 2). Mature females were significantly larger than mature males within each river (p < 0.001 for each).

**Table 1:** Year class lengths and frequency distribution of *Pteronarcys californica* larvae collected 30 April- 1 May 2010 from the Colorado and Fraser Rivers. Lengths in mm from anterior tip if head to posterior tip of epiproct.

Year Class	Male larvae (n = 149)	Female larvae (n = 123)
1	≤15 mm (33)	≤17 mm (41)
2	16–25 (34)	18–25 (25)
3	26–34 (31)	26–38 (29)
4	≥ 35 (51)	≥ 39 (28)

**Table 2:** Year class lengths and frequency distribution of *Pteronarcys californica* larvae collected 14 April 2010 from the Gunnison River. Lengths in mm from anterior tip if head to posterior tip of epiproct.

Year Class	Male larvae (n = 191)	Female larvae (n = 129)
1	≤23 mm (162)	≤23 mm (95)
2	24–36 (14)	24–40 (12)
3	≥37 (15)	≥41 (22)

Exuvia densities were highly correlated with both mature larval densities (R^2^ = 0.90) and total larval densities (R^2^ = 0.88). Exuvia densities averaged 2.6/m^2^ and ranged from 0.002/m^2^ to 11.443/m^2^ (Table 3). Total larval density averaged 80.0/m^2^ and ranged from 0 to 437.3/m^2^. Density of mature larvae averaged 16.1/m^2^ and varied from 0 to 101.3/m^2^. While there was a strong relationship between larvae density and exuvia density, the relationship was not 1:1. Larval estimates were generally higher than exuvia estimates except at sites two, three, and seven where exuvia were found but no larvae. To predict the density of mature larvae, the linear equation was: larval density = 7.358*(exuvia density) - 2.854.

**Table 3:** Densities in m^2^ of *Pteronarcys californica* pre-emergent larvae, all larvae, total exuvia counted, and three pass exuvia population estimates from the Huggins Closed Capture model collected from April-June 2010 from the Colorado, Fraser, and Gunnison Rivers.

Site	Densities m^2^			
	All larvae	Pre-emergent larvae	Exuvia	Exuvia population estimate
1	8.00	1.33	0.854	0.872
2	0.00	0.00	0.064	0.065
3	0.00	0.00	0.077	0.079
4	4.00	4.00	2.391	2.477
5	4.00	1.33	0.686	0.697
6	44.00	4.00	0.306	0.315
7	0.00	0.00	0.002	0.002
8	5.33	0.00	0.007	0.007
9	297.33	101.33	11.001	11.443
10	437.33	49.33	9.392	9.849

Exuvia surveys detected *P*. *californica* populations at all 10 sites whereas larvae were found at only seven of the 10 sites in benthic samples (Table 3). Capture probabilities of exuvia with the two pass model ranged from 0.33 to 0.92 (average 0.73). Capture probabilities with the three pass Huggins model ranged from 0.43 to 0.87 (average 0.70) for the first pass and 0.38 to 0.85 (average 0.62) on the second pass. The time varying capture probability model was the favored by AIC*c* over the constant capture probability in nine of the nineteen surveys and had an average model weight of 0.54 vs. 0.47 for the two pass model.

## Discussion

**Fig 2:**
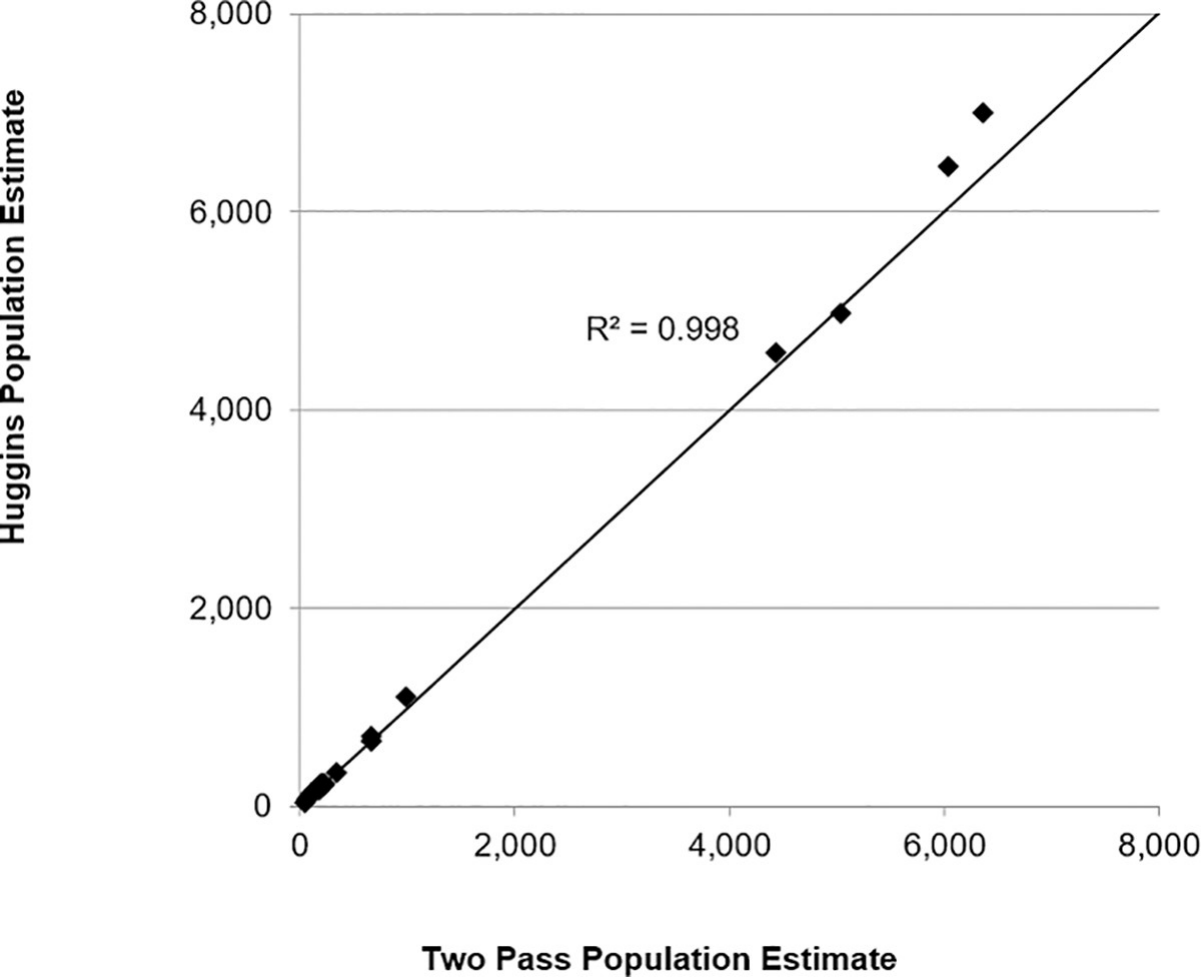
Relationship of population estimates from a three pass Huggins Closed Capture model in Program MARK (with time effect that allowed for varying capture probabilities) and the simple two pass model of that assumes a constant capture probability.

Multiple pass removal estimates of *P*. *californica* exuvia effectively predicted densities of total larvae as well as mature late instar larvae. While there was evidence that capture probabilities did vary by pass in some of the surveys (47%), any bias in the population estimates appears to be small. Population estimates derived from the Huggins model and the two pass model were highly correlated by the coefficient of determination and population estimates were extremely close by the two methods (Fig 2). The average difference between population estimates from the two methods was 7% and ranged from 0–25%. Our work agrees with previous analysis in both fish and dragonflies that for removal sampling to give accurate estimates, capture probabilities should be as high as possible and/or more than two passes should be used [32, 37]. Capture probability of exuvia declined at low density sites and extremely high density sites. For sites with moderate densities of exuvia (214–994 per 30.5 m) capture probabilities estimated by the Huggins model averaged 0.78 and the difference in population estimates by the two methods averaged only 4%, indicating that two pass method was sufficient at these sites. The estimated capture probability for second and third passes was estimated to higher than the first pass in five of the 19 surveys indicating that the bias in population estimates due to differing capture probabilities was not in a consistent direction as reported with fish [37]. Assumptions of the multiple pass depletion models appeared to be met well at moderate density sites. The two-pass depletion technique performed well at these sites due to immobility of exuvia, high capture probability, and the lack of size selective gear [36, 37], suggesting two sampling passes can be adequate if three or four passes are cost prohibitive as with Odonata exuvia [32].

Correlation between densities of exuvia and all larval age classes were high but not 1:1. Estimates of exuvia density from removal surveys underestimated benthic larvae at high density sites but overestimated larvae at low density sites. The underestimation of larval densities is likely due to the behavior of mature larvae congregating near the river bank prior to emergence in the shallower water where benthic sampling with a Surber sampler occurs. Further work is necessary with different sampling techniques to evaluate the spatial distribution of the larvae pre-emergence. The underestimation of larvae by benthic sampling at low density sites was likely due to the high capture probability of exuvia in conjunction with the known difficulty of collecting the larvae of rare invertebrates using a Hess or Surber sampler [44]. The exuvia sampling technique effectively reduces large areas of available benthic habitat to smaller, well defined, and more easily accessible riparian sampling areas. Riparian sampling areas averaged 61 m^2^ (30.5 m long x 2 m wide). They represented 742 m^2^ (400–1500 m^2^) of unevenly distributed benthic habitat, much of which was not accessible by wading due to excessive water depth and velocities and could not be effectively sampled with traditional benthic sampling equipment like Hess or Surber samplers. Therefore, exuvia sampling may more accurately estimate larval densities than benthic sampling at low density sites and be better for detecting rare invertebrate species with the emergence behaviors and conspicuous exuvia that allow for removal sampling.

Presence of exuvia or adults is the only evidence of successful life cycle completion. Varying densities can indicate habitat quality and help identify reference sites and priority areas for river conservation, restoration, and monitoring of *P*. *californica*. In regions where *P*. *californica* does not occur, this technique may be useful for other easily recognizable stoneflies like *Pteronarcella badia*, *Claassenia sabulosa*, *Hesperoperla pacifica*, or mayflies like *Timpanoga hecuba*. This technique eliminates the need for benthic sample collection, preservation, and subsequent expense of processing in the laboratory. It also provided accurate and less biased density estimates of *P*. *californica* larvae than those derived from benthic samples.

Benthic sampling of aquatic invertebrates is a useful and productive biomonitoring technique but the overall process to acquire data can be labor and cost intensive. In addition, it can be difficult to find target species that are rare at a site with benthic sampling [44]. Using multiple pass removal sampling of the recently shed exuvia can be an effective and efficient way to estimate densities of *P*. *californica* and may be superior to traditional benthic sampling at detecting the species at very low densities.

## References

[1] Goodnight CJ. The use of aquatic macroinvertebrates as indicators of stream pollution. Transactions of the American Microscopical Society. 1973; 92: 1–13. 4735832

[2] Rosenberg DM, Resh VH. Introduction to freshwater biomonitoring and benthic macroinvertebrates. Chapman and Hall, New York 1993.

[3] Barbour MT, Gerritsen J, Snyder BD, Stribling JB. Rapid bioassessment protocols for use in streams and wadeable rivers: periphyton, benthic macroinvertebrates and fish. 2nd edition U.S. Environmental Protection Agency; Office of Water; Washington, D.C 1999

[4] Elder JA, Gaufin AR. Notes on the occurrence and distribution of Pteronarcys californica Newport (Plecoptera) within streams. Great Basin Naturalist. 1973; 33: 218–220.

[5] Surdick RF, Gaufin AR. Environmental requirements and pollution tolerance of Plecoptera. Environmental Protection Agency, Environmental Monitoring and Support Laboratory, Cincinnati, Ohio 1978.

[6] Kauwe JSK, Shiozawa DK, Evans RP. Phylogeographic and nested-clade analysis of the stonefly *Pteronarcys californica* (Plecoptera: Pteronarcyidae) in the Western USA. Journal of the North American Bentholgical Society. 2004; 23: 824–838.

[7] Myers LW, Kondratieff BC. Larvae of North American species of *Pteronarcys* (Plecoptera: Pteronarcyidae). Illiesia. 2017, 13:192–224. 10.25031/2017/13.16

[8] Fore LS, Karr JR, Wisseman RW. Assessing invertebrate responses to human activities: Evaluating alternative approaches. Journal of the North American Benthological Society. 1996; 15: 212–231.

[9] Freilich JE. Movement patterns and ecology of *Pteronarcys* nymphs (Plecoptera): observations of marked individuals in a Rocky Mountain stream. Freshwater Biology. 1991; 25: 379–394.

[10] Needham JG, Claassen PW. A Monograph of the Plecoptera of North America North of Mexico. Entomological Society of America, Lafayette, Indiana 1925.

[11] Smith L. Studies of North American Plecoptera (Pteronarcinae and Perlodini). Transactions of the American Entomological Society. 1917; 43: 433–489. Available from: http://www.jstor.org/stable/25076980

[12] Gaufin AR, Ricker ER, Miner M, Milam P, Hays RA. The stoneflies (Plecoptera) of Montana. Transactions of the American Entomological Society. 1972; 98: 1–161.

[13] Brusven MA, Prather KV. Influence of stream sediment on distribution of macrobenthos. Journal of the Entomological Society of British Columbia. 1974; 71: 25–32.

[14] Heinold BD. The mayflies (Ephemeroptera), stoneflies (Plecoptera), and caddisflies (Trichoptera) of the South Platte River Basin of Colorado, Nebraska, and Wyoming. M. Sc. Thesis, Colorado State University. 2010. 10.13140/RG.2.2.24657.17761

[15] DeWalt RE, Stewart KW. Life histories of stoneflies (Plecoptera) in the Rio Conejos of southern Colorado. Great Basin Naturalist. 1995; 55: 1–18.

[16] Townsend GD, Pritchard G. Larval growth and development of the stonefly *Pteronarcys californica* (Insecta: Plecoptera) in the Crowsnest River, Alberta. Canadian Journal of Zoology. 1998; 76: 2274–2280.

[17] Sheldon AL. Emergence patterns of large stoneflies (Plecoptera: Pteronarcys, Calineuria, Hesperoperla) in a Montana river. Great Basin Naturalist. 1999; 59: 169–174.

[18] Fuller RL, Stewart KW. Stonefly (Plecoptera) food habits and prey preference in the Dolores River, Colorado. American Midland Naturalist. 1979; 101: 170–181.

[19] Short RA, Maslin PE. Processing of leaf litter by a stream detritivore: Effect on nutrient availability to collectors. Ecology. 1977; 58: 935–938.

[20] Erickson RC. Benthic field studies for the Windy Gap study reach, Colorado River, Colorado, fall 1980 to fall 1981. Prepared for The Northern Colorado Water Conservancy District, Municipal Sub-District. 1983.

[21] Nehring RB. Stream fisheries investigations. Colorado Division of Wildlife, Federal Aid in Sportfish Restoration, Project F-51-R, Progress Report, Fort Collins. 1987.

[22] Needham JG, Christenson RO. Economic insects in some streams of northern Utah. Logan, UT: Utah Agricultural Experiment Station Bulletin. 1927; 201: 1–36.

[23] Baxter CV, Fausch KD, Saunders WC. Tangled webs: reciprocal flows of invertebrate prey link streams and riparian zones. Freshwater Biology. 2005; 50: 201–220. 10.1111/j.1365-2427.2004.01328.x

[24] Muttkowski RA. The food of trout in Yellowstone National Park. Roosevelt Wild Life Bulletin. 1925; 2: 470–497.

[25] Vinson M. A short history of *Pteronarcys californica* and *Pteronarcella badia* in the Logan River, Cache County, Utah. Utah State Bug Lab. Pteronarcyidae History Ongoing blog. Last updated 14 January 2008. Available from: https://www.usu.edu/buglab/Content/Files/salmonfly%20history.pdf

[26] Birrell J, Nelson CR. Loss of the Giant Salmonfly *Pteronarcys californica* and changes in stonefly diversity in the Provo River, Utah (Plecoptera). Journal of Undergraduate Research, Brigham Young University. 2018 Available from: http://jur.byu.edu/?p=23170

[27] Stagliano DM. Evaluation of salmonflies in Montana's rivers: are statewide populations really declining? Montana Natural Heritage Program, Helena 2010. 29 pp.

[28] Nehring RB, Heinold BD, Pomeranz JF. Colorado River aquatic resources investigations. Colorado Division of Wildlife, Federal Aid Project F-237R-18, Final Report, Fort Collins. 2011. Available from: https://www.academia.edu/26701834/Colorado_River_Invertebrate_Investigations?auto=download

[29] Ruse LP. Chironomid community structure deduced from larvae and pupal exuvia of a chalk stream. Hydrobiologia. 1995; 315: 135–142.

[30] Foster SE, Soluk DA. Evaluating exuvia collection as a management tool for the federally endangered Hine’s emerald dragonfly, *Somatochlora hineana* Williamson (Odonata: Cordulidae). Biological Conservation. 2004; 118: 15–20.

[31] Raebel EM, Merckx T, Riordan P, Macdonald DW, Thompson DJ. The dragonfly delusion: why it is essential to sample exuvia to avoid biased surveys. Journal of Insect Conservation. 2010; 14: 523–533.

[32] DuBois RB. Detection probabilities and sampling rates for Anisoptera exuvia along river banks: influences of bank vegetation type, prior precipitation, and exuvia size. International Journal of Odonatology. 2015; 18: 205–215.

[33] Richards DC, Rolston MG, Dunkel FV. A comparison of salmonfly density upstream and downstream of Ennis Reservoir. Intermountain Journal of Sciences. 2000; 6: 1–9.

[34] Zippin C. The removal method of population estimation. Journal of Wildlife Management 1958; 22: 82–90.

[35] Cooch E, White G. Program MARK: A Gentle Introduction. 17th edition. 2017. Available from: http://www.phidot.org/software/mark/docs/book/.

[36] Peterson JT, Thurow RF, Guzevich JW. An evaluation of multipass electrofishing for estimating the abundance of stream-dwelling salmonids. Transactions of the American Fisheries Society. 2004; 133: 462–475.

[37] Riley SC, Fausch KD. Underestimation of trout population size by maximum likelihood removal estimates in small streams. North American Journal of Fisheries Management. 1992; 12: 768–776.

[38] Branham JM, Hathaway RR. Sexual differences in the growth of Pteronarcys californica Newport and *Pteronarcella badia* (Hagen) (Plecoptera). Canadian Journal of Zoology. 1975; 53: 501–506.

[39] Schultheis AS, Bootha JY, Vinson MR, Miller MP. Genetic evidence for cohort splitting in the merovoltine stonefly *Pteronarcys californica* (Newport) in Blacksmith Fork, Utah. Aquatic Insects. 2008; 187–195.

[40] Huggins RM. On the statistical analysis of capture-recapture experiments. Biometrika. 1989; 76:133–140.

[41] White GC, Burnham KP. Program MARK: survival estimation from populations of marked animals. Bird Study. 1999; 46:sup1: S120–S139.

[42] Burnham KP, Anderson DR. Model selection and multimodel inference: a practical information-theoretic approach. 2nd ed New York: Springer-Verlag; 2002.

[43] R Development Core Team. R: A language and environment for statistical computing. 2012 R Foundation for Statistical Computing, Vienna, Austria URL http://www.R-project.org/.

[44] Vinson MR, Hawkins CP. Effects of sampling area and subsampling procedure on comparisons of taxa richness among streams. Journal of the North American Benthological Society. 1996; 15: 392–399.

